# Quantitative Proteomics Reveals the Beneficial Effects of Low Glucose on Neuronal Cell Survival in an *in vitro* Ischemic Penumbral Model

**DOI:** 10.3389/fncel.2020.00272

**Published:** 2020-09-01

**Authors:** Hua Li, Farooqahmed S. Kittur, Chiu-Yueh Hung, P. Andy Li, Xinghong Ge, David C. Sane, Jiahua Xie

**Affiliations:** ^1^Department of Pharmaceutical Sciences, Biomanufacturing Research Institute and Technology Enterprise, North Carolina Central University, Durham, NC, United States; ^2^Department of Dermatology, General Hospital of Ningxia Medical University, Yinchuan, China; ^3^Carilion Clinic, Virginia Tech Carilion School of Medicine, Roanoke, VA, United States

**Keywords:** neuronal cells, ischemic penumbra, hypoxia, low glucose, proteomic analysis

## Abstract

Understanding proteomic changes in the ischemic penumbra are crucial to rescue those salvageable cells and reduce the damage of an ischemic stroke. Since the penumbra region is dynamic with heterogeneous cells/tissues, tissue sampling from animal models of stroke for the molecular study is a challenge. In this study, cultured hippocampal HT22 cells under hypoxia treatment for 17.5 h with 0.69 mM low glucose (H+LG) could mimic ischemic penumbral cells since they had much higher cell viability and viable cell number compared to hypoxia without glucose (H−G) treatment. To validate established cell-based ischemic penumbral model and understand the beneficial effects of low glucose (LG), quantitative proteomics analysis was performed on H+LG, H−G, and normoxia with normal 22 mM glucose (N+G) treated cells. We identified 427 differentially abundant proteins (DAPs) between H−G and N+G and further identified 105 DAPs between H+LG and H−G. Analysis of 105 DAPs revealed that LG promotes cell survival by activating HIF1α to enhance glycolysis; preventing the dysregulations of extracellular matrix remodeling, cell cycle and division, and antioxidant and detoxification; as well as attenuating inflammatory reaction response, protein synthesis and neurotransmission activity. Our results demonstrated that this established cell-based system could mimic penumbral conditions and can be used for molecular studies.

## Introduction

**Figure 1:**
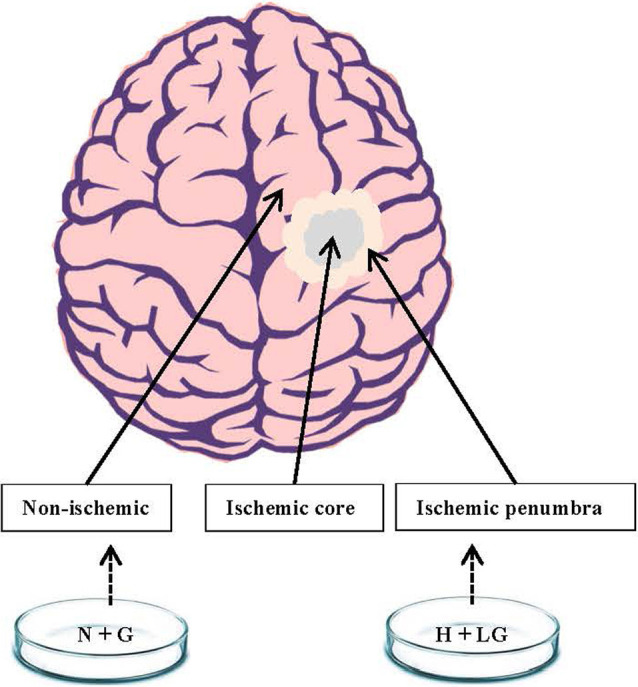
A schematic diagram showing ischemic penumbra and non-ischemic regions of the brain. We postulated that HT22 cells under hypoxia with low glucose (H+LG) for 17.5 h treatment could mimic the cells in the ischemic penumbra region while cells under normoxia with 22 mM glucose (N+G) treatment would be similar to non-ischemic cells.

Stroke is the second leading cause of death and the third biggest cause of disability worldwide, with ischemic stroke accounting to 87% of the cases (Feigin et al., 2017; Puig et al., 2018). Because of its clinical significance and socioeconomic burden, extensive studies have been conducted to reveal the pathogenic mechanism(s) of hypoxic-ischemic brain injury. Despite many efforts, ischemic damage remains one of the leading causes of chronic disability or death owing to lack of efficient treatments (Iadecola and Anrather, 2011; Mergenthaler et al., 2013; Moretti et al., 2015; Benjamin et al., 2017; McCabe et al., 2018; Huang and Zhang, 2019). In cerebral ischemia, occlusion of a cerebral artery causes decreased blood flow and impaired delivery of oxygen, glucose, and nutrients to brain tissues, which lead to the formation of ischemic core, ischemic penumbra and non-ischemic regions based on cerebral blood flow (CBF) gradient (Figure 1; Olsen, 2009; Robbins and Swanson, 2014; McCabe et al., 2018). Cells within the ischemic core are irreversibly damaged, and cannot be rescued. The ischemic penumbra is an area around the ischemic core at which cells are absent of induced electrical potentials but metabolically viable and potentially salvageable (Astrup et al., 1977; Symon et al., 1977; Fisher and Bastan, 2012). However, these cells could die if not rescued promptly. Since they are potentially salvageable, the ischemic penumbra has been the main target of acute therapeutic interventions (Olsen, 2009; Robbins and Swanson, 2014; Chamorro et al., 2016; McCabe et al., 2018). Enhanced understanding of the cellular and molecular events associated with ischemic penumbra is essential to identify the therapeutic target(s) and develop therapeutic strategies.

Various animal models of focal cerebral ischemia have been created to investigate pathophysiological changes in the ischemic penumbra. Each model has its advantages to address certain specific questions but with limitations (McCabe et al., 2018). The major limitation is animal tissue sampling because the penumbral region is unstable and dynamic with both regional and temporal fluctuations in blood flow (Obrenovitch, 1995), and the brain tissues within the region are physiologically heterogeneous (Shiraishi et al., 1989; McCabe et al., 2018). To study pathophysiological changes occurring in the penumbra, using an *in vitro* cell-based model with homogenous cells could be an alternative. *In vitro* cell models to mimic hypoxia-ischemia by combined oxygen and glucose deprivation (OGD) have been used to investigate the molecular mechanisms of pathophysiological changes in response to hypoxic-ischemic damage (Newell et al., 1995; Hillion et al., 2005; Datta et al., 2009; Meloni et al., 2011; Tasca et al., 2015). However, the previous *in vitro* cell models under OGD conditions may not mimic ischemic penumbra well because the culture does not include glucose. In the penumbral region, it is known that it is reduced but non-zero CBF between non-ischemic and ischemic tissues (Robbins and Swanson, 2014; McCabe et al., 2018). Therefore, both oxygen and glucose are critical in cerebral ischemia and should be considered in developing an *in vitro* penumbral model.

The importance of continuous glucose supply for normal brain physiology and function has been well documented since neurons have the highest energy demand while lacking glycogen stores (Marty et al., 2007; Mergenthaler et al., 2013; Robbins and Swanson, 2014). When cerebral ischemia occurs, the delivery of glucose and oxygen is impaired, which causes ATP depletion and in turn triggers dysregulation of numerous processes leading to cell death. Glucose rapidly entering anerobic metabolism is important to generate ATP for cell survival (Robbins and Swanson, 2014) at the cost of producing lactic acid, which reduces the pH of brain tissues and exacerbates brain injury (Ying et al., 1999; Xiong et al., 2004). Glucose is also required for both quenching and production of reactive oxygen species (ROS) in the central nervous system (Bhardwaj et al., 1998; Suh et al., 2008; Mergenthaler et al., 2013).

Additionally, the ischemic penumbral region experiences a limited supply of oxygen, and cells in this region are hypoxic. The cellular response to hypoxia has been well studied in tumors (Majmundar et al., 2010; Carnero and Lleonart, 2016). However, how neuronal cells within the ischemic penumbra respond to the hypoxic conditions remains elusive even though the regulation of glucose metabolism to protect both neurons and cancer cells from hypoxia-induced apoptosis was found to be similar (Mergenthaler et al., 2013). In tumors, the HIF family of transcription factors has been identified as the main mediators of cellular response to hypoxia (Guzy et al., 2005; Majmundar et al., 2010). Among HIF transcription factors, HIF1 that comprises of a constitutively expressed β subunit and an oxygen-dependently α subunit plays key roles in adaptive responses of cells to hypoxic stress (Semenza, 2009). HIF1α is hydroxylated by prolyl hydroxylases (PHDs) to be degraded under oxygen-sufficient conditions but would be stable under hypoxic conditions owing to low enzymatic activities of PHDs (Semenza, 2009). Under hypoxia, HIF1α is translocated to the nucleus and induces expression of a large number of genes from multiple pathways and biological processes (Benita et al., 2009; Slemc and Kunej, 2016). Among HIF1α targeted genes, the largest group is associated with glucose uptake and metabolism as reported in tumor cells/tissues under hypoxia (Gatenby and Gillies, 2004; Denko, 2008; Majmundar et al., 2010; Carnero and Lleonart, 2016). Further protein profiling in the ischemic penumbra region responding to the hypoxic conditions may allow us to discover the underlining pathways associated with cell survival and death in this region.

Recent advances in quantitative proteomic techniques have made it possible to profile the comprehensive protein expression levels more precisely and reproducibly (Hu et al., 2016). Several studies focusing on the cellular proteomic changes based on either hypoxia alone or OGD treatment in neuronal cells provided some valuable information to understand how OGD influences cellular changes and contributes to the neuronal damage and death (Jin et al., 2004; Datta et al., 2009; Zhou et al., 2011; Herrmann et al., 2013). However, only limited proteins were identified and most of the OGD models were based on tumor-derived neuronal cells (Jin et al., 2004; Datta et al., 2009; Zhou et al., 2011; Herrmann et al., 2013; Djidja et al., 2014; Qi et al., 2014; Gao et al., 2017). To mimic ischemic penumbra, using neuronal cells of non-tumor origin for quantitative proteomic analysis might allow us better to identify the ischemic stress-induced pathways for developing therapeutic interventions.

In the present study, we subjected mouse hippocampal homogeneously HT22 cells to H+LG and N+G treatment to mimic the ischemic penumbra and non-ischemic region (Figure 1), respectively with H−G (OGD) treatment as a control. Hippocampus, as a vital center for learning and memory, is extremely vulnerable to ischemic insults compared with other brain parts in both animals and humans (Schmidt-Kastner and Freund, 1991; Fujioka et al., 2000; Nakatomi et al., 2002; Barth and Mody, 2011). Therefore, HT22 is a suitable cell line for the present study. The addition of a mere 0.69 mM glucose improved the cell survival compared to H−G treatment for 17.5 h, indicating that low glucose (LG) promotes cell survival under hypoxic conditions. To understand cellular and molecular events occurring in HT22 cells subjected to hypoxia with or without glucose, we applied label-free one-dimensional liquid chromatography, tandem mass spectrometry (1D-LC-MS/MS) to study proteomic changes.

## Materials and Methods

### Cell Culture, and Glucose-Hypoxia Treatments

HT22 cells were cultured in Hyclone DMEM high glucose (22 mM) medium (GE Healthcare) supplemented with 2 mmol/L L-glutamine, 100 IU/ml penicillin, 0.1 mg/ml streptomycin, and 10% FBS (Corning) at 37°C under 5% CO_2_. For studying the effects of H−G treatment on cell viability, 1 × 10^6^ cells in 6 ml DMEM medium were seeded onto a 60 mm petri dish. After culturing for 24 h in a CO_2_ incubator until the cell reaches 80% ~ 90% confluence, DMEM medium was replaced with glucose-free Neurobasal™-A medium (Thermo Fisher Scientific) with the same supplements as above, and the uncovered Petri dishes were placed in a hypoxia chamber (STEMCELL Technologies Inc.) for oxygen deprivation treatment. The hypoxia chamber was purged with 95% N2:5% CO_2_ for 5 min to displace oxygen, then sealed and placed in a CO_2_ incubator at 37°C for 0, 4.5, 11, 17.5, or 24 h. Under this oxygen deprivation treatment, the cultures might have low levels of oxygen to mimic ischemic penumbral conditions despite the exact levels in the medium were not determined due to the device’s limitation. HT22 cells with DMEM high glucose medium were set up as an N+G control. After treatments, cells were detached with trypsin and cell viability was measured by a Trypan blue dye exclusion method using a Beckman Coulter Vi-CELL XR cell viability analyzer (Beckman Coulter). To identify a culture condition to mimic the ischemic penumbra, DMEM high glucose medium was mixed with glucose-free Neurobasal™-A medium to reach 22, 11, 5.5, 2.75, 1.38, 0.69, or 0 mM glucose concentrations to determine their effects on cell survival under hypoxic conditions. Hypoxia treatment and cell viability measurements were carried out as described above.

To prepare samples for proteomic analysis, 2.8 × 10^6^ cells in 15 ml medium were seeded on a 100 mm petri dish. They were grown under normoxia or hypoxia with or without 0.69 mM glucose for 17.5 h. Following treatments, cells were collected and cell viability was measured. The remaining cells were collected and washed with cold PBS thrice and stored at −80°C for proteomics, transcriptional expression, and immunoblotting analyses. For HIF1α immunoblotting, new batches of cells were prepared after they were cultured for 4.5 and 17.5 h. Cells were harvested immediately after aspirating media and quickly washed with cold PBS and then lysed directly to minimize HIF1α degradation by reducing air exposure.

### Quantitative Proteomic Analysis

Quantitative proteomic analysis was performed at the Duke Proteomics and Metabolomics Shared Resource. Sample preparation, quantitative mass spectrometry analysis, and MS/MS data analysis and search were performed following the methodologies described by Foster et al. (2017) with a few differences. Briefly, cells were lysed by sonication in 100 μl buffer of 0.5% acid-labile surfactant (ALS1) followed by heating at 80°C for 5 min with additional sonication to fully denature DNA. Protein concentrations were measured by Bradford assay and 25 μg of each sample was reduced with 10 mM dithiothreitol at 80°C for 5 min. After cooling, samples were alkylated with 25 mM iodoacetamide at room temperature in the dark for 30 min. Next, excess iodoacetamide was quenched with 10 mM dithiothreitol before proteins were digested with 1:25 (w/w trypsin/protein) Sequencing Grade Modified Trypsin (Promega) at 37°C overnight. To inactivate trypsin and to degrade ALS1 after digestion, samples were acidified with 1% trifluoroacetic acid and 2% MeCN, followed by heating to 60°C for 2 h. After centrifugation, a QC pool was made by mixing equal quantities of all samples, and samples were transferred to Maximum Recovery LC vials (Waters Corporation).

Quantitative 1D-LC-MS/MS was performed on 750 ng of the peptide digests per sample in singlicate with additional analyses of conditioning runs and QC pools as described in Supplementary Table S1. Samples were analyzed using a nanoACQUITY ultraperformance liquid chromatography-tandem mass spectrometry (UPLC) system (Waters Corporation) coupled to a Fusion Lumos high-resolution accurate mass tandem mass spectrometer (Thermo Fisher Scientific) *via* a nanoelectrospray ionization source. The sample was first trapped on a Symmetry C18 180 μm × 20 mm trapping column (5 μl/min at 99.9/0.1 v/v H_2_O/MeCN). Next, an analytical separation was implemented using a 1.7 μm AQCUITY HSS T3 C18 75 μm × 250 mm column (Waters, Milford, MA, USA) with a 90 min gradient of 5–30% MeCN with 0.1% formic acid at a flow rate of 400 nl/min and column temperature of 55°C. Data collection on the Fusion Lumos MS was accomplished in data-dependent acquisition mode with a 240,000 resolution (@ *m*/*z* 200) full MS scan from *m*/*z* 375–1,600 with a target AGC value of 2e5 ions and 50 ms maximum injection time with internal calibration enabled. Peptides were selected for MS/MS using advanced peak determination enabled, peptide monoisotopic peak determination, and including charge states 2–5. MS/MS used HCD fragmentation and detection in the ion trap with following settings: an isolation width of 0.7 m/z; a normalized collision energy of 30 ± 5%; a rapid ion trap scan rate; maximum AGC of 5e3 ions; maximum injection time of 300 ms and use of all available parallelization time. A 20 s dynamic exclusion was enabled. The total analysis cycle time for each sample analysis was approximately 2 h.

Following UPLC-MS/MS analyses, data were imported into Rosetta Elucidator v.4 (Rosetta Biosoftware Inc.), and analyses were aligned based on the accurate mass and retention time of detected ions (“features”) using PeakTeller algorithm in Elucidator. Relative peptide abundance was calculated based on area-under-the-curve (AUC) of the selected ion chromatograms of the aligned features across all runs. The proteome had 375,136 quantified features and 1,247,473 higher collision energy (peptide fragment) spectra. The MS/MS data were searched against a Swissprot database *Mus musculus* taxonomy (downloaded on 09/05/17) with additional common contaminants and standards (e.g., bovine serum albumin) and an equal number of reverse entries for decoy database searching (w/ 33,839 total entries). Mascot Distiller and Mascot Server (v 2.5, Matrix Sciences) were utilized to produce fragment ion spectra and to perform the database searches. Database search parameters included precursor mass tolerance of 5 ppm, product ion mass tolerance of 0.6 Da, trypsin specificity with up to two missed cleavages, fixed modification on Cys (carbamidomethyl) and variable deamidation (N/Q) and acetylation (protein *N*-terminus). After individual peptide scoring using the PeptideProphet algorithm in Elucidator, the data was annotated at a 0.8% peptide false discovery rate (FDR; PeptideTeller score of >0.6). For quantitative processing, the data was first curated to contain only high-quality peptides with appropriate chromatographic peak shape by eliminating those low-quality peptides with any abnormal shapes, such as shoulder peaks, split peaks, and peak broadening and the dataset was intensity scaled to robust mean as described by Feger et al. (2016). Obtained proteins, which were quantified across all samples, were further filtered with a criterion for higher confidence of identification and quantification by first removing those proteins quantified with just 1 peptide, then using a ProteinTeller score of >0.87 (equating to a 1% protein FDR) as a cutoff before they were used for downstream analysis.

### Database Enrichment and Network Analysis

Differentially abundant proteins (DAPs) between H−G and N+G treatments were selected by 1.4-fold change (*p* ≤ 0.05) as a threshold. To screen the LG responsive proteins under hypoxia conditions, the DAPs in H+LG and H−G were first selected by 1.4-fold change (*p* ≤ 0.05) compared with N+G treatment as a threshold, respectively. Then they were further compared to identify DAPs between H+LG and H−G with the fold-change cutoff ≥1.5 as a threshold. Abundance-increased or -decreased proteins were classified by gene ontology (GO) analysis based on biological process, cellular component, and molecular function using the WebGestalt platform^1^ (Liao et al., 2019), and the parameters used for the enrichment analysis were the minimum of 5 and maximum of 2,000 IDs in the category, as well as the Benjamini Hochberg (BH) method with the FDR ≤ 0.05. Also, the KEGG pathway enrichment was analyzed using KOBAS 3.0 platform^2^ (Wu et al., 2006) while STRING web (version 10.5^3^, Szklarczyk et al., 2015) was used to query about the interactions of DAPs as well as enrichment of GO terms and KEGG pathways. ClustVis web^4^ tool (Metsalu and Vilo, 2015) was used for heat map visualization.

### Gene Expression and Immunoblotting Analyses

RNeasy Mini Kit (QIAGEN) was used for total RNA isolation and *β-actin* used as an internal control. Five biological replicates were used, and gene expressions were measured following the method described previously (Hung et al., 2010). Briefly, a High-Capacity cDNA Reverse Transcription Kit (Applied Biosystems) was used to synthesize the first-strand cDNA. Then the qRT-PCR was conducted using Power SYBR Green PCR Master Mix (Applied Biosystems) with gene-specific primers employing a QuantStudio™ 6 Flex Real-Time PCR system (Applied Biosystems). A relative expression level of each gene was calculated first by comparing it to the *β-actin* in the same sample to obtain a ΔCt value. Then the fold change of transcript abundance of each gene between two samples was calculated by comparing their ΔCt values to obtain ΔΔCt in which one ΔΔCt value (2^−ΔΔCT^) represents two-fold change. Data from five sets of biological samples were averaged. All primers are listed in Supplementary Table S2.

For immunoblotting, total proteins were extracted using M-Per reagent containing Halt Protease and Phosphatase Inhibitor Cocktail (Thermo Fisher Scientific). For nuclear protein extraction, Qproteome Cell Compartment Kit (Qiagen) was used. Protein separation and band signal detection were conducted as previously described (Zimmerman et al., 2018). Briefly, the protein lysates (about 20 μg per well) were separated using 4–12% Bis-Tris NuPAGE gels (Invitrogen) and then transferred to PVDF membranes using a Bio-Rad Mini Trans-Blot system. After transfer, membranes were blocked in a 1:1 solution of Li-COR Odyssey Blocking buffer (Li-COR, Inc.) and PBS. The membranes were subsequently probed with the indicated primary antibodies (see below) and then with IRDye Infrared Dyes conjugated secondary antibodies (Li-COR, Inc.) before image acquisition by the Li-COR Odyssey Classic Imaging System. The fluorescent signals were quantified and analyzed with the Li-COR image studio software version 5.2.5, and the target protein signals among samples were normalized to each loading control. Antibodies recognizing GTR1/GLUT1 (#12939S, 1:1,000, Cell Signaling), NSA2 and RPB2/POLR2B (#A14475 and #A5928, 1:1,000, ABclonal), HIF1α (#NB100-105, 1:500, Novus Biologicals), LOXL3 (#sc-377216, 1:500, Santa Cruz Biotechnology), RPB1/POLR2A (#39097, 1:1,000, Thermo Scientific), beta-actin (#A2228, 1:5,000, Sigma-Aldrich), and Histone H3 (#3638, 1:1,000, Cell Signaling) were used. Beta-actin or Histone H3 was used as an internal control. IRDye 680RD conjugated to Goat anti-Rabbit IgG or IRDye 800CW conjugated to Goat-anti-Mouse IgG (LI-COR Biosciences) was used as secondary antibodies.

### Statistical Analysis

For all experiments except proteomic analysis, obtained results were subjected to statistical analysis using one-way ANOVA followed by Fisher’s least significant difference for multiple comparisons and Student’s *t*-test for pairwise mean comparison (*p* < 0.05). For proteomic analysis, statistical tests were performed as described by Foster et al. (2017).

## Results

### The Response of HT22 Cells to Hypoxia With and Without Glucose Treatment

**Figure 2:**
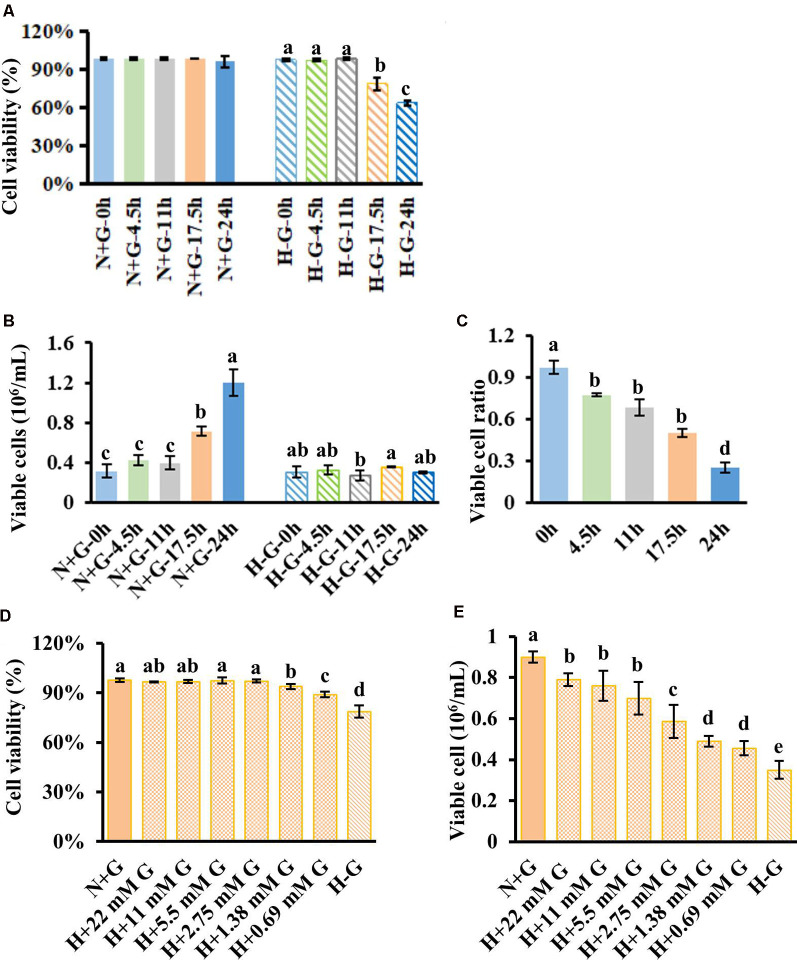
HT22 cell viability and viable cell number under hypoxia along with different concentrations of glucose treatments. Cell viability **(A)** and viable cell number **(B)** with its ratio **(C)** measured at different time points under normoxia with 22 mM glucose (N+G) and hypoxia without glucose (H−G). Cell viability **(D)** and viable cell number **(E)** of HT22 cells subjected to hypoxia for 17.5 h with different concentrations of glucose along with N+G and H−G treatments. The viable cell ratio was calculated using viable cells in H−G divided by those in H+G at the same time point. All data plotted are the average of three independent experiments ± SD. One-way ANOVA followed by Fisher’s least significant difference for multiple comparisons was used for statistical analysis. Different letters labeled represent significant differences at *p* < 0.05 level among treatments.

To develop an *in vitro* penumbral model, we tried to establish a cell culture system with a low level of glucose under hypoxic conditions for a certain period at which cells maintain relatively high viability but dramatically reduce viable cell numbers (VCNs) compared to N+G treatment. To this end, we first investigated the culture response of HT22 cells under H−G conditions to find a time point at which cell viability is significantly impacted but potentially could be recovered by glucose supply. Therefore, cells were cultured under H−G and N+G conditions for 0–24 h to measure the cell viability and VCNs. Results showed that H−G treatment for 17.5 and 24 h caused ~20% and ~30% decrease in both cell viability (Figure 2A), respectively. Furthermore, the total numbers of viable cells following H−G treatment were similar for all tested time points but were much higher in N+G treatment after cultured for 17.5 and 24 h (Figure 2B). Thus, the ratios of total viable cells between H−G and N+G treatments were reduced to 22%, 32%, 44%, and 71% from 4.5 to 24 h (Figure 2C). These results indicate that H−G treatment affected cell division and/or growth. The impact of H−G treatment on cell viability and cell number was limited at 11 h whereas 24 h treatment was too severe. To ensure that the treated cells could potentially recover from the H−G treatment, the 17.5 h with ~20% reduced cell viability, and 45% VCN was selected as a suitable time point to study the impact of glucose on cell survival.

We then tested beneficial effects of glucose from a normal high concentration of 22 mM to as low as 0.69 mM under hypoxia for 17.5 h. Results showed that as low as 0.69 mM glucose could significantly recover cell viability to ~89% from ~79% of H−G treatment while 1.38 mM and above concentrations had similar cell viabilities to that of N+G treatment (Figure 2D). Under 22 mM glucose, there was no difference in cell viability in the presence or absence of oxygen (Figure 2D), but VCN was significantly reduced under hypoxia (Figure 2E). Supplementing 0.69 mM glucose increased VCN by ~30% compared to H−G treatment (Figure 2E), indicating that glucose as low as 0.69 mM benefited HT22 cell survival and growth under hypoxia conditions but some cell death occurred. This situation is akin to ischemic penumbra where sub-regions of high and low CBF exist (Ginsberg, 2003) with low cerebral blood glucose exhibiting some cell death depending on the duration of hypoxia (Le Feber et al., 2016). Therefore, this culture condition with 17.5 h hypoxia plus 0.69 mM glucose could better represent the penumbral-like condition and was designated as H+LG for the following analyses.

### Quantitative LC-MS/MS Analysis

**Figure 3:**
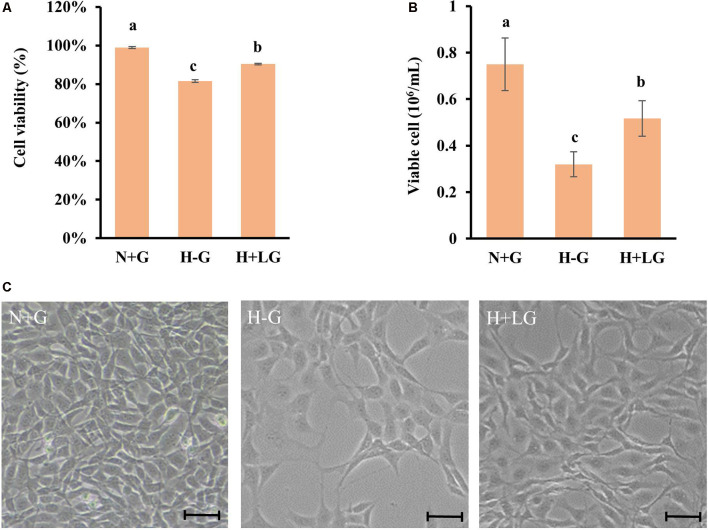
HT22 cells under N+G, H−G, and H+LG for 17.5 h. Cell viability **(A)** and viable cell number **(B)** were plotted as the average of three independent experiments ± SD. One-way ANOVA followed by Fisher’s least significant difference for multiple comparisons was used for statistical analysis. Different letters labeled represent significant differences at *p* < 0.05 level among treatments. Images of cell density and morphology **(C)** were recorded under a Nikon Eclipse Ts2 Inverted Routine Microscope. Scale bar = 100 μm.

To investigate the beneficial effects of LG under hypoxia at molecular levels, proteomic changes in cells subjected to H+LG for 17.5 h were compared with the cells exposed to N+G and H−G as positive and negative controls, respectively. Cell viability (Figure 3A) and VCN (Figure 3B) of three different treatments were confirmed as previously observed in Figures 2A,B. Reduced cell densities of H−G and H+LG treatments were further confirmed by microscopic observation (Figure 3C). After establishing a penumbra-like condition, quantitative proteomics analysis was performed on cells exposed to N+G, H−G, and H+LG. Using UPLC-MS/MS analysis, a total of 48,603 peptides (Supplementary Table S3) and 5,409 proteins (Supplementary Table S4) were quantified across all samples. Of these, 4,012 proteins were quantified by 2 or more peptides. Among them, 3,837 had a ProteinTeller score of >0.87 (equating to a 1% protein FDR; Supplementary Table S5), which were used for downstream analyses. For biological variability, the percent coefficient of variations (%CVs) was measured for each protein across the individual analyses. The mean %CV of the QC pool was 7.8% for all proteins and 4.9% for proteins quantified by 2 or more peptides. The variability of the biological samples was 20.3% for all proteins and 15.7% for proteins quantified by 2 or more peptides. These results reveal that the technical reproducibility was good and the biological variability was low.

### Proteomic Changes Under OGD Conditions

**Figure 4:**
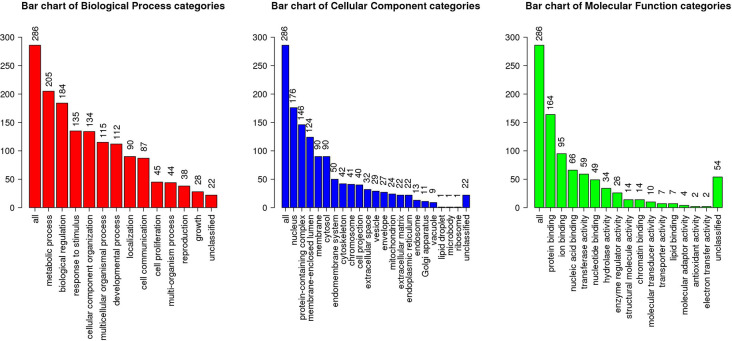
Gene ontology (GO) slim summary of abundance decreased differentially abundant proteins (DAPs) between H−G and N+G treatments. Over-representation analysis (ORA) results of the biological process, cellular component, and molecular function were presented. The heights of the bars represent the numbers of user list genes observed in the category.

**Figure 5:**
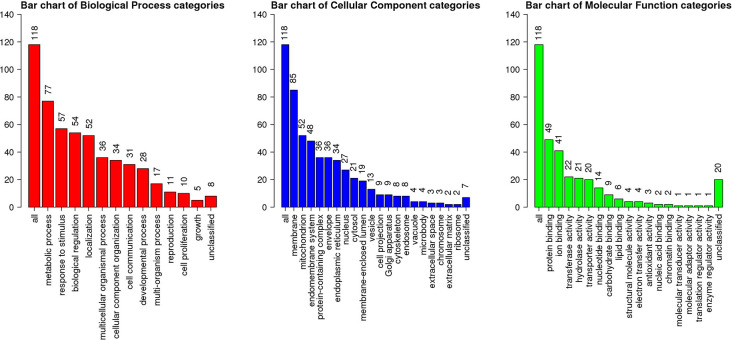
GO slim summary of abundance increased DAPs between H−G and N+G treatments. ORA results of biological process, cellular component, and molecular function were presented. The heights of the bars represent the numbers of user list genes observed in the category.

Among 3,837 proteins, a total of 427 DAPs with 304 abundance-decreased and 123 abundance-increased between 17.5 h H−G and N+G treatments (Supplementary Table S6) was identified with a 1.4-fold change (*p* ≤ 0.05) used as a threshold to define DAPs. Among 427 DAPs, 404 could be assigned to different GO terms. The results of over-representation analysis (ORA) were presented in Figures 4, 5. In the biological process, 118 abundance-increased and 286 abundance-decreased DAPs were enriched in metabolic process, response to the stimulus, biological regulation, cellular component organization, localization, multicellular organismal process, and six other categories. The ratios of abundance-decreased to -increased DAPs are around 2–3 times in 10 out of 12 enriched categories while more than four times in the categories of cell proliferation and growth. The results of enrichment analysis in biological pathways (Supplementary Table S7) also showed inhibitory effects of H−G treatment on cell division and growth. The abundance-decreased DAPs were largely enriched in response to transcription, cell division, and growth while the abundance-increased DAPs were mainly enriched in response to oxygen levels or ischemia, pyruvate metabolic process, generation of energy and metabolites, and fatty acid metabolism (Supplementary Table S7). These proteomic changes with more abundance-decreased DAPs in cell proliferation and growth correlated well with observed low cell viability and VCN after H−G treatment (Figures 2, 3). The most decreased DAPs of these two categories were PEDF, PR2C2, and IL6RB, which are known to play key roles in neuronal survival and growth (Araki et al., 1998; Wang et al., 2006; Yang et al., 2016).

Among cellular component categories, more abundance-increased than abundance-decreased DAPs were enriched in the mitochondrion, envelope, endoplasmic reticulum, and microbody, implying that these organelles responded strongly to H−G stress. As for molecular function, the majority of DAPs in enzyme regulator activity and nucleic acid binding categories were abundance-decreased while ratios between abundance-decreased and abundance-increased DAPs in most remaining categories are ~2–3 times. Interestingly, all nine DAPs (Supplementary Table S8) in the carbohydrate-binding category were abundance-increased, which are involved in glycolysis, and *N-* and *O-*glycosylation, suggesting that cells enhanced glucose metabolism and/or protein glycosylation to adapt to OGD stress.

**Figure 6:**
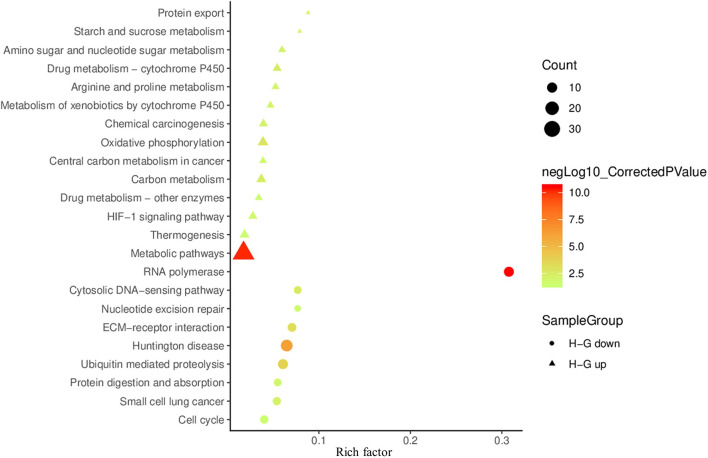
Scatter plot of KEGG enrichment results of DAPs between H−G and N+G treatments. The up-regulated and down-regulated enrichment pathways are shown in the bubble chart with more than three genes enriched and the corrected *p* < 0.05. The rich factor is the number ratio of annotated DAPs compared to all annotated genes in the same pathway. The larger value means the greater degree of pathway enrichment. The colors of the circles (down) or triangles (up) represent in the −log_10_ ranges (corrected *p*-value) where the higher value indicates greater pathway enrichment.

To determine affected cellular pathways, 427 DAPs were also mapped in the KOBAS 3.0 and performed KEGG pathway enrichment analysis. We identified 14 enriched pathways for abundance-increased and nine for abundance-decreased DAPs (Figure 6). A large number of abundance-increased DAPs were enriched in metabolic pathways in agreement with GO analysis results. Among the abundance-increased KEGG pathways, the HIF1 signaling pathway was enriched because HIF1 is a hypoxia-inducible transcription factor (Guzy et al., 2005; Majmundar et al., 2010). Moreover, the oxidative phosphorylation pathway related to ATP production (Senior, 1988) was also enriched. Among the abundance-decreased KEGG enriched pathways, RNA synthesis, cell cycle, and ECM-receptor interaction were enriched, which might be responsible for the observed reduced cell viability and VCNs under H−G treatment.

### Beneficial Effects of LG Are Through Several Mechanisms

**Figure 7:**
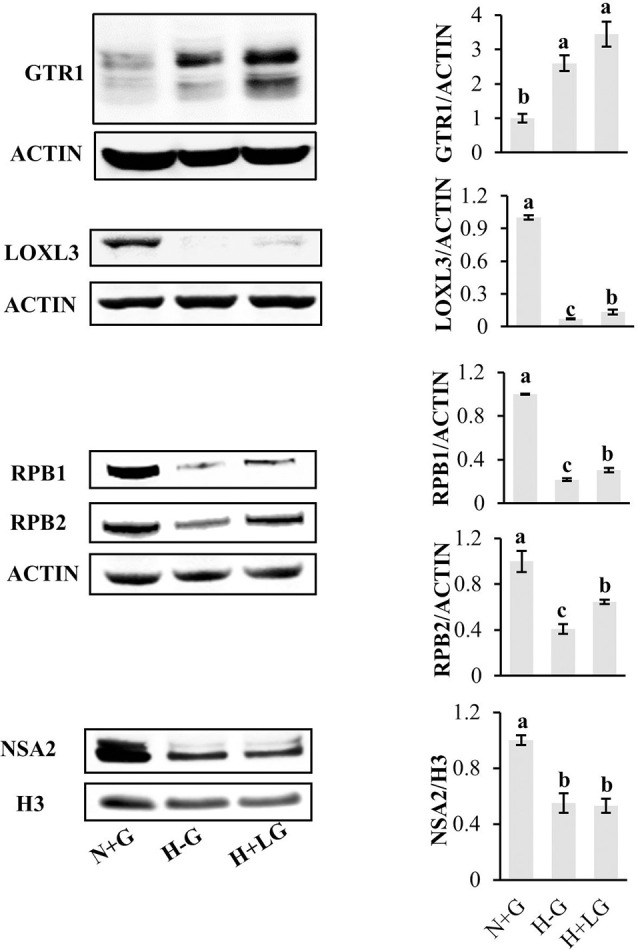
Western blot analysis of five selected proteins from HT22 cells treated by three different conditions. Representative western blots (left) and quantification analysis (right) of GTR1, LOXL3, RPB1, RPB2, and NSA2 are shown. The band intensity of five selected proteins from Western blotting was calculated against either actin or histone 3 (H3) on the same blot. The ratio was further calculated against the N+G treatment whose relative expression level was set as 1. All data plotted are the average of three independent experiments ± SD. One-way ANOVA followed by Fisher’s least significant difference for multiple comparisons was used for statistical analysis. Different letters labeled represent significant differences at *p* < 0.05 level among treatments.

To reveal LG beneficial effects for cell survival under hypoxia conditions, the protein abundance profiles between H+LG and H−G treatments were compared using N+G treatment as the control. With differentiated abundance change cutoff ≥1.5-fold, a total of 105 (81 abundance-increased and 24 abundance-decreased) DAPs were identified (Supplementary Table S9). Since these DAPs were identified from H+LG treated cells with higher viability and VCN (Figure 3), they must be glucose-responsive DAPs, and their abundance changes could provide some valuable information concerning the beneficial effects of LG. Before conducting further analysis, we validated the proteomic results by randomly selecting five proteins GTR1, LOXL3, RBP1, RBP2, and NSA2 (Supplementary Tables S6, S9) to perform immunoblotting. Immunoblotting results showed that the expression level of GTR1 was significantly induced while those of the remaining four proteins were significantly suppressed by H−G treatment for 17.5 h compared to H+G treatment (Figure 7), which is consistent with proteomic data. In H+LG treated cells (Figure 7), their expression patterns except for NSA2 also matched well with our proteomic data. Therefore, the 105 DAPs were further subjected to heat map analysis using the ClutVis web tool.

### Survival Benefit of LG Is Through the Activation of HIF1α and Enhancement of Glycolysis

**Figure 8:**
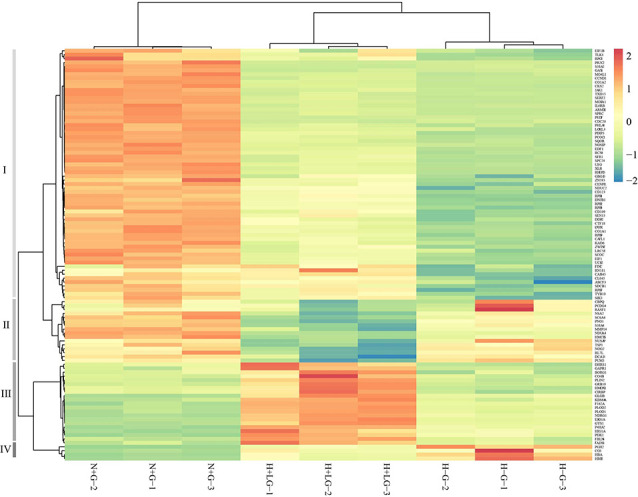
Heat map from hierarchical clustering of 105 DAPs between H−G and H+LG treatments. Abundances of these 105 proteins from N+G treated cells were also included for clustering analysis. DAPs are clustered into four groups (I to IV) based on abundance similarity. The color key scale bar at the upper right shows Z-score values for the heat map.

Heat map results of 105 DAPs showed that they could be classified into four groups (Figure 8). What intrigued us most are the proteins clustered in the group III with 21 proteins (Supplementary Table S10), which all showed significantly higher abundances in H+LG than H−G or N+G treated cells (Supplementary Table S9). Eight of them had ≥1.5-fold higher abundances in H−G than N+G treated cells. All of these also had nearly 2-fold higher abundances in H+LG than H−G treated cells. Also, these eight (HIG1A, PDK1, P4HA2, NDRG1, GYS1, GLGB, ERO1A, and PLOD2) with three additional DAPs (F162A, PLOD1, and HMOX1) in this group are hypoxia-responsive and targeted by HIF1α (Benita et al., 2009; Czibik et al., 2011; Gilkes et al., 2013; Wang et al., 2013; Ameri et al., 2015; Wu et al., 2019).

**Figure 9:**
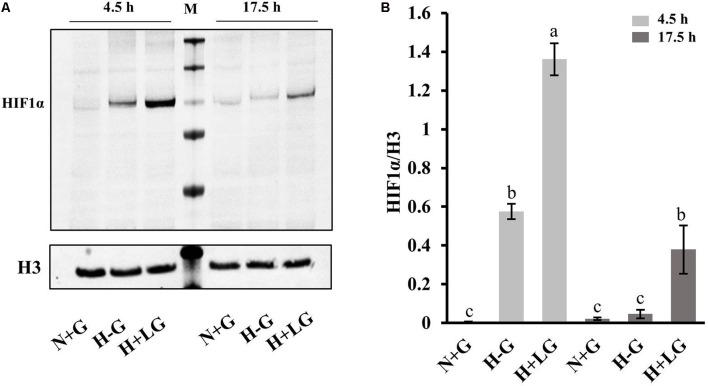
Western blot analysis of HIF1α. **(A)** Representative Western blotting of nuclear proteins isolated from cells treated with N+G, H−G, or H+LG conditions for 4.5 or 17.5 h against anti-HIF1α and control anti-histone 3 (H3). **(B)** The ratio of band intensity between HIF1α and H3 was calculated. All data plotted are the average of three independent experiments ± SD. One-way ANOVA followed by Fisher’s least significant difference for multiple comparisons was used for statistical analysis. Different letters labeled represent significant differences at *p* < 0.05 level among treatments.

Considering the higher abundances of the above HIF1α targeted proteins in H+LG and H−G treated cells, we suspected that the expression levels of HIF1α might be higher. In proteomics analysis, no HIF1α was detected presumably being degraded during cell harvesting and downstream processing under oxygen-rich conditions because it is sensitive to oxygen (Semenza, 2009). To prevent degradation, we prepared three new batches of cells, and quickly harvested cells and extracted nuclear proteins as described in “Experimental Procedures” to avoid exposure to oxygen. Since HIF1α accumulates in the nucleus under hypoxia conditions (Semenza, 2009) and its accumulation could occur earlier than 17.5 h of hypoxia treatment, we used nuclear fractions isolated from both 4.5 h and 17.5 h treated cells to measure its accumulation levels. Immunoblotting results showed that HIF1α accumulated after 4.5 h in both H−G and H+LG treated cells, but not in N+G treated cells at either time point (Figure 9). Moreover, HIF1α was detected in H+LG treated cells, but barely in H−G treated cells after 17.5 h culture (Figure 9). These results indicate that HIF1α was induced under hypoxia conditions and LG could promote its stability, which supports the idea that above-identified hypoxia-responsive DAPs were very likely induced by HIF1α.

Regarding the functions of the above eight proteins, overexpression of NDRG1 has been shown to result in more proliferation and less apoptosis in cancer cells (Susanne et al., 2004; Ellen et al., 2008; Wang et al., 2013; Said et al., 2017). Hypoxia-induced expression of HIGD1A can increase oxygen consumption and ATP synthesis benefiting cell survival (Hayashi et al., 2015). Under hypoxic conditions, knocking down ERO1A (Takei et al., 2017) and PLOD2 (Song et al., 2017) affects cell proliferation. The reduction of P4HA2 expression was also reported to inhibit cell proliferation (Xiong et al., 2014). The remaining three are involved in glycolysis and glycogen metabolism, which are necessary for optimal glucose utilization to adapt to OGD stress (Saez et al., 2014). The induction of GYS1 and GLGB has been shown to correlate well with an increase in glycogen synthase activity and glycogen accumulation in cells exposed to hypoxia (Pescador et al., 2010; Favaro et al., 2012). PDK1 is an important enzyme activated by HIF1α to suppress the tricarboxylic acid cycle to rescue hypoxia-induced apoptotic cells through increasing ATP levels and attenuating hypoxic ROS generation (Jung-Whan et al., 2006). All these hypoxia-responsive proteins have positive contributions to cellular adaptation to hypoxia stress.

**Figure 10:**
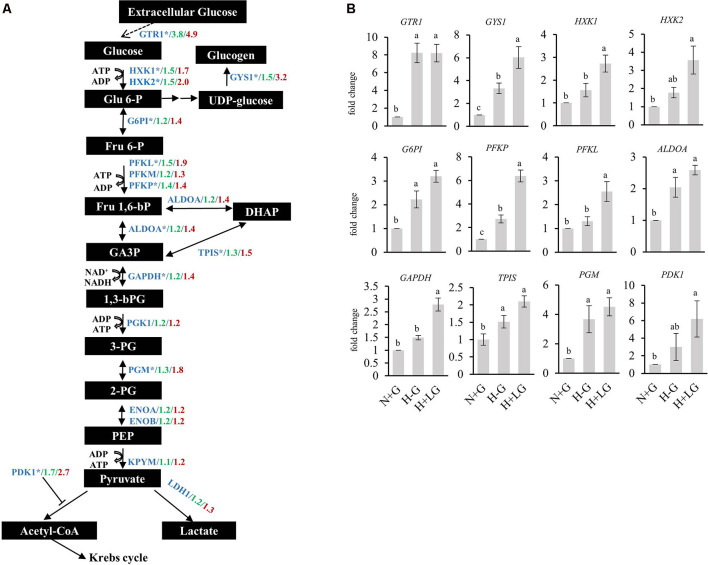
Expressions of proteins and genes involved in glucose metabolism under N+G, H−G, and H+LG treatments. **(A)** A schematic diagram of the glucose metabolic pathway was adapted from Hamanaka and Chandel (2012) and Leiherer et al. (2014). All metabolites were labeled in black boxes, while proteins were marked in blue. The levels of fold changes between H−G and N+G from the quantitative proteomic analysis were in green, while those between H+LG and N+G were in red. **(B)** The results of qRT-PCR of selected genes from the glucose metabolic pathway marked by an asterisk in **(A)**, which was based on abundance change between H+LG and N+G treatments ≥1.4-fold. Fold changes were calculated by comparing to N+G after being normalized with *β-actin* and set as 1. All data plotted are the average of five independent experiments ± SD. One-way ANOVA followed by Fisher’s least significant difference for multiple comparisons was used for statistical analysis. Different letters labeled represent significant differences at *p* < 0.05 level among treatments.

Furthermore, activation of glycolytic/gluconeogenic pathway genes by HIF1 is considered as a critical metabolic adaptation to hypoxia through the strengthened conversion of glucose to pyruvate and consequently to lactate (Xiong et al., 2004; Fulda and Debatin, 2007; Denko, 2008; Benita et al., 2009; Düvel et al., 2010; Majmundar et al., 2010; Slemc and Kunej, 2016). This consideration prompted us to examine the glycolysis/gluconeogenesis related enzymes from our proteomic data further. Most of the detected glycolytic pathway-related enzymes were abundance-increased under hypoxia conditions, and their abundances were higher in the H+LG compared to H−G treated cells (Figure 10). Next, we performed qRT-PCR analysis to investigate transcriptional levels of those genes in the glycolytic pathway whose protein abundances induced by 0.69 mM glucose ≥1.4-fold compared to N+G treatment. Consistent with the increase in the abundances of DAPs observed in the proteomics study, we found that all 12 selected genes were up-regulated under H−G conditions and were even higher in H+LG treated cells (Figure 10). These results indicate that induced expression of these glycolysis/gluconeogenesis pathway genes is important for cell survival under hypoxic conditions.

### Survival Benefit of LG Is Through Attenuation of OGD Induced Detrimental Effects

The group I with 59 proteins had their abundances significantly lower in H−G treated cells compared to N+G cells (Supplementary Table S10). Under H+LG treatment, about three-fourths of them could be partially recovered and one-fourth of them were fully recovered (Supplementary Table S9). Proteins in this group are known to play important roles in ECM remodeling, transcriptional/translational activities, cell cycling and division, cell survival pathway, inhibition of apoptotic activity, ubiquitination and autophagy activities, antioxidant and detoxification, and cell differentiation (Supplementary Table S10). Previous studies show that ECM remodeling is critical for cell adaptation under stress like hypoxia (Pankov and Yamada, 2002; Gilkes et al., 2013; Riis et al., 2016), and proteins like fibronectin in this pathway were reported to promote neural stem cell survival and cell proliferation (Tate et al., 2002; Jan and Richard, 2010). Partial or complete restoration of an abundance of these proteins involved in transcription, translation, and cell survival and division pathways in H+LG treatment, therefore, indicates that LG prevents the dysregulation of multiple cellular pathways essential for cell survival and growth.

### Survival Benefit of LG Is Also Through Adjusting Over-Response of Inflammatory Reaction Proteins to OGD Stress

Group IV is composed of four proteins whose abundance increased dramatically following H−G treatment, but their H−G induced abundances reduced nearly 2-fold by LG (Supplementary Tables S9, S10). These proteins are involved in acute inflammatory reactions. Neuronal hemoglobin α and β chains (HBA and HBB2), and complement protein CO3 that are known to be strongly induced following ischemia (Cowell et al., 2003; Mocco et al., 2006; Alawieh et al., 2015; Codrich et al., 2017) were also found to be induced strongly following H−G treatment in our study. Furthermore, the abundance of prostaglandin G/H synthase 2 (PGH2), which is rapidly induced in inflamed tissues and its reaction products are thought to contribute to ischemic brain damage (Seibert et al., 1994; Nogawa et al., 1998), was also found to be increased following H−G. The fact that the presence of 0.69 mM glucose reduced the abundance of these pro-inflammatory proteins by 2-fold indicates that it exerts anti-inflammatory response. Adjusting over-response of these inflammatory reaction proteins to OGD induced stress could have beneficial effects on cell survival.

### Survival Benefit of LG Is Also Through Lowering Protein Synthesis and Neurotransmission Activity

In group II, there are 20 proteins with their abundance changes ≥ 1.5-fold between H+LG and H−G treatments (Supplementary Tables S9, S10). However, their change patterns are different from the remaining groups. Compared to N+G, 13 out of 20 DAPs were not affected by H−G treatment but were reduced ≥1.5-fold by H+LG treatment. The other seven DAPs had their abundances reduced 1.5–52-fold by H−G treatment while they were further reduced 1.5- to 4-fold by H+LG. Since higher cell viability and VCN were observed in H+LG than H−G treated cells, understanding this group of proteins with these special patterns of abundance changes is essential. Five of them (PNO1, DCA13, NSA2, NOG2, and RL7L) have functions in ribosomal biogenesis and PUM3 serves as a translational regulator of specific mRNAs by binding to their 3’ untranslated regions (Chang et al., 2011). Suppression of these proteins under H+LG treatment suggests that lowering protein synthesis is also one of the survival strategies because protein translation demands high energy. The cells under low energy and oxygen have been reported to use alternative transcription pathways and translation machinery to synthesize only essential proteins to promote cell survival (Chee et al., 2019) even though its regulatory mechanism is unclear. Also, two proteases (MMP14 and CBPQ) and four proteins which promote apoptosis [RASF1 (Oh et al., 2006), NUSAP (Nie et al., 2010), GAS1 (Mellström et al., 2002) and TSP1 (Rege et al., 2009)] were also reduced. With LG, further reduction of the abundances of these proteins is believed to benefit cell survival.

Additionally, it is interesting to note that four of this group (PCDG4, SC6A6, S38A4, and S38A2; Table 1; Supplementary Table S10) are involved in neurotransmission activities and had their abundances reduced by LG. S38A2 and S38A4 are two sodium-coupled neutral amino acid transporters and S38A2 regulates the amino acid pool as well as the recycling of the neurotransmitter (Hatanaka et al., 2000, 2001; Angelina et al., 2011) while SC6A6 is sodium-dependent taurine and beta-alanine transporter as well as a neurotransmitter transporter (Tomi et al., 2008). It has been demonstrated that hypoxia diminishes the expression and function of system A (alanine-preferring) amino acid transporters (Nelson et al., 2003). Since electrical inactivity but sufficient to preserve ion channels of the neurons is one of the features of ischemic penumbra (Astrup et al., 1977; Symon et al., 1977), further lowering expression levels of these proteins related to neurotransmission might be another way to contribute to cell survival. This also led us to search neurotransmission activity-related proteins in remaining DAPs between H−G and N+G treated cells (Supplementary Table S6) to study how they responded to H+LG treatment. Seven proteins involved in neurotransmission activities were found to be significantly inhibited by H−G treatment compared to N+G treatment, but their expressions were not further affected by LG (Table 1). Having these two groups of proteins affected by H−G might further affect or not by LG supply suggests that LG cannot improve electrical failure.

**Table 1:** Effects of low glucose on proteins involved in neurotransmission activity*.

Protein name	Protein description	H−G/N+G	*p*-value	H+LG/N+G	*p*-value
Proteins with their abundances reduced by low glucose supply
PCDG4	Protocadherin gamma-A4	1.08	0.644	−1.50	0.021
SC6A6	Sodium- and chloride-dependent taurine transporter	−1.41	0.070	−2.42	0.007
S38A4	Sodium-coupled neutral amino acid transporter 4	−1.36	0.073	−3.44	0.016
S38A2	Sodium-coupled neutral amino acid transporter 2	−20.05	0.014	−30.38	0.006
Proteins with their abundances not further affected by low glucose supply
NPTXR	Neuronal pentraxin receptor	−2.43	0.018	−2.36	0.008
NCOR2	Nuclear receptor corepressor 2	−2.36	0.019	−1.72	0.019
CDNF	Cerebral dopamine neurotrophic factor	−2.36	0.007	−2.19	0.011
NRP1	Neuropilin-1	−1.61	0.031	−2.12	0.008
NRP2	Neuropilin-2	−6.25	0.002	−7.88	0.01
ESYT1	Extended synaptotagmin-1	1.41	0.010	1.56	0.015
UPAR	Urokinase plasminogen activator surface receptor	−10.22	0.001	−7.03	0.003

*Selected differentially abundant proteins (DAPs) involved in neurotransmission activity either H+LG vs. N+G or H−G vs. N+G.

### The Possible Connection of the 105 DAPs Between H+LG and H−G Treatments

**Figure 11:**
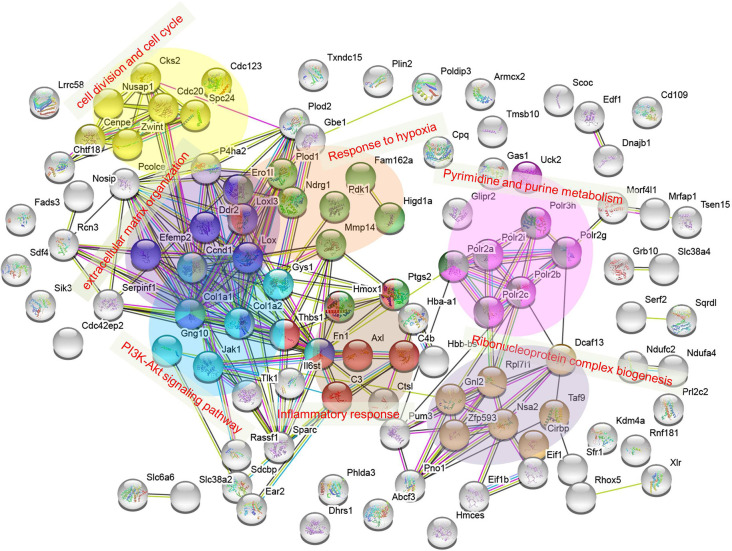
Protein interaction network for 105 DAPs listed in Supplementary Table S9. The interaction network was obtained using the STRING database http://string-db.org/ with a PPI enrichment *p* < 1.0 × 10^−16^. Clustered proteins were indicated by different colors. Categories of pyrimidine and purine metabolism and PI3K-Akt signaling pathway were assigned from functional KEGG pathway analysis, while extracellular matrix organization, inflammatory response, cell division, and cell cycle, and ribonucleoprotein complex biogenesis were from GO term enrichment.

To find out the possible connection of the above 105 DAPs between H+LG and H−G treatments, a string web version for protein and protein interaction with GO and KEGG pathway analysis were employed for further analysis. The proteins were mainly clustered into seven groups, which are pyrimidine and purine metabolism, PI3K-AKT signaling pathway, cell division and cell cycle, extracellular matrix organization, response to hypoxia, inflammatory response, and ribonucleoprotein complex biogenesis, respectively (Figure 11). The results of this analysis agree with the above heat map analysis results that LG influences many pathways to benefit cell survival.

## Discussion

In the present study, we established an HT22 cell culture condition with H+LG for 17.5 h to mimic ischemic penumbral cells under which cells had better survival performance compared with H−G. It is known that in penumbra region, cells are viable but devoid of electrical potential because of the inhibition of proteins involved in cell division and proliferation (Demyanenko and Uzdensky, 2017) and ion-channel proteins that generate electrical potential (Astrup et al., 1977; Symon et al., 1977; Fisher and Bastan, 2012). Ischemic stroke is also known to favor glycolysis in the ischemic penumbra and thus to promote pyruvate production and lactate accumulation (Arnberg et al., 2015; Xu et al., 2017). Additionally, there is accumulating evidence from experimental and clinical studies supporting the existence of an inflammatory reaction in the ischemic penumbra (Gauberti et al., 2016). The above described molecular changes in the ischemic penumbra were also confirmed by the proteomic changes in our proteomic data. Our proteomic data revealed that H−G treatment mainly inhibited metabolism, cell division processes, protein synthesis, and neurotransmitter activity, while induced hypoxia response, glycolysis and inflammatory reactions compared to N+G treatment. When these H−G induced changes were further compared to those in H+LG treated cells, LG supply was found to enhance glycolysis *via* HIF1α activation, attenuate H−G induced inflammatory reaction and regulate other cells survival/death-related pathways, but depress protein synthesis and neurotransmission activity. Overall, our proteomic results demonstrate that established H+LG for 17.5 h culture system shared certain features found in the ischemic penumbra, which could be used as an *in vitro* penumbral model for molecular studies.

To get insight into pathophysiological changes in the ischemic penumbra, it is desirable to investigate its translational changes. With quantitative proteomic analysis, we detected 3,837 proteins qualified with high confidence (Supplementary Table S5), which is much larger than previous cell-based studies in which 524–1081 proteins were identified under OGD treatments (Jin et al., 2004; Datta et al., 2009; Zhou et al., 2011; Herrmann et al., 2013). Importantly, 427 and 398 proteins in our study had their abundance changed ≥1.4-fold (*p* < 0.05) between H−G or H+LG and N+G treatments, respectively, and 105 proteins had their abundance difference ≥1.5-fold between H−G and H+LG treated cells. This large number of identified DAPs allowed us to cluster them in functional groups and pathways and provided us a better opportunity to understand the relationship among proteomic changes, oxygen levels, glucose levels, and cell survival.

The most important finding is the survival benefit of LG through several mechanisms. Primarily, it occurs through HIF1α-mediated enhancement of glycolysis. Previous studies have shown that neurons and cancer cells both depend exclusively on glucose metabolism to generate ATP for their energy, and the former was suggested to use similar mechanisms as the latter to adapt to substrate deprivation and promote survival (Mergenthaler et al., 2013). In tumors, hypoxia can develop within the tumor mass to activate HIF1α because of impaired vascularization. Activated HIF1α then induces the expression of glucose transporters (GTR1 and GTR3) and some enzymes in the glycolytic pathway (Carnero and Lleonart, 2016). In HT22 cells, we also found that HIF1α was stabilized and accumulated in cells under H−G conditions and LG supply would further stabilize HIF1α (Figure 9), which is consistent with previous reports (Vordermark et al., 2005). Our proteomics data also showed an increase in the abundances of some of the glycolysis related proteins and glucose transporter GTR1 by hypoxia and LG (Figure 10; Supplementary Tables S6). Increases in the abundance of PDK1 in H+LG treated cells compared to H−G further suggested that glucose metabolism in these cells was diverted from the TCA cycle to glycolysis to produce ATP and minimize the effect of ROS (Jung-Whan et al., 2006). Thus, we believe that the survival benefit of LG supply is possibly through HIF1α-mediated enhancement of glycolysis.

Also, LG appears to benefit cell survival by other mechanisms. The observed decrease in the abundances of pro-inflammatory proteins HBA, HBB2, CO3, and PGH2, suggests that the survival benefit of LG could be associated with attenuating OGD induced inflammatory reaction. Slowing down high-energy consuming activities, such as protein biosynthesis (group II in Supplementary Table S10) and neurotransmission (Table 1), maybe additional mechanisms for the LG to benefit cell survival. Furthermore, the decrease in the abundances of cell-death promoting proteins (group I) and the increase in the abundances of cell survival and proliferation proteins (HIG1A, P4HA2, ERO1A, PLOD1, NDRG1 and group I proteins; Supplementary Table S10) also suggest that LG promotes cell survival *via* directly regulating these pathways.

Can the observed survival benefit of LG supply to neuronal cells under hypoxic conditions be exploited for any therapeutic practice? During brain ischemia, residual glucose supports glycolytic ATP production even in the absence of oxygen (Shirato et al., 2010; Robbins and Swanson, 2014). The idea of glucose protection on *in vitro* neuronal cells was recognized several decades ago (Schurr et al., 1987; Callahan et al., 1990). Unfortunately, using intravenous infusion to increase circulating blood glucose was found to have harmful effects (Pulsinelli et al., 1982; Wass and Lanier, 1996; MacDougall and Muir, 2011). A correlation between elevated admission glucose concentrations and poor outcomes was always observed in clinical practice (Bellolio et al., 2014). However, strict glycaemic control failed to yield any beneficial outcome (Wan Sulaiman et al., 2014; Johnston et al., 2019).

The observed protective effects of LG in the current study and previously reported adverse effects of glucose admission after stroke and reperfusion injury led us to think about how glucose can have opposite effects. Rat cerebral extracellular glucose concentration is only 1/3rd of that in the blood (Silver and Erecińska, 1994). Cerebral glucose concentrations drop sharply when brain CBF levels fall, suggesting that the extracellular glucose level in the ischemic penumbra should be lower than the non-ischemic region, and well below that in blood. Theoretically, glucose supply could compensate ischemia-caused diminishing extracellular glucose levels in the ischemic penumbra and subsequently promote the salvageability of cells. Additionally, hyperglycemia was found in 30–60% of all stroke patients with most of them non-diabetic (Capes et al., 2001; Bruno et al., 2014). This high percentage of non-diabetic stroke patients with hyperglycemia might also imply that quickly elevating blood glucose levels is a possible mechanism to compensate for lower glucose in ischemic tissues to promote cell survival. The observed harmful effects of glucose intervention on stroke patients could be caused by elevated blood glucose concentrations to produce detrimental side effects. During ischemia, the blood-brain barrier is disrupted (Lin et al., 2015) and elevated circulating glucose concentrations could worsen the barrier function (Huang et al., 1996). Previous studies have also demonstrated that hyperglycemia increases endothelial protein kinase C activation, amplifies inflammatory responses and increases superoxide generation, resulting in increased edema formation and hemorrhage and decreased microvascular reflow (Huang et al., 1996; Cipolla et al., 2011; Won et al., 2011; Robbins and Swanson, 2014). All these changes can contribute to cerebral vascular injury. Thus, elevating circulating glucose concentrations is not a good choice for intervention. To supply ischemic penumbral cells with additional glucose for their survival, on-site delivery of glucose to this region may be helpful and practical, and worth to be tested in stroke animal models.

In conclusion, our study has demonstrated that LG has beneficial effects on HT22 cell survival under hypoxic conditions, which was supported by our proteomic results. Given that timely rescue of ischemic penumbral cells is critical to lessen stroke consequences, finding survival benefits of LG is valuable, which might be developed into a therapeutic approach. However, further validation of current findings from HT22 cells in primary hippocampal neurons with more functional parameter measurements or directly in ischemic penumbra brain tissues is essential to fully understand the cellular and molecular events associated with ischemic penumbra. Additionally, using the same type of basic culture medium in future validation study is critical since culture media used in the present study for N+G, H−G and H+LG treatments were two types or mixed. Although the majority of the components in these two media are the same, any differences between the two types of media might introduce some variations in the results. Because of this limitation, the major conclusions in the present study were carefully drawn from the comparison between cells grown from H−G (glucose-free Neurobasal A medium) and H+LG (96.9% glucose-free Neurobasal A medium + 3.1% DMEM with high glucose) media, which led us to believe that the observed expression differences in genes and proteins between H−G and H+LG treated cells are more likely influenced by the glucose instead of other ingredients. Nevertheless, the *in vitro* ischemic penumbral model established can assist future studies to dissect affected pathways, identify therapeutic targets, and test any therapeutic approaches.

## Data Availability Statement

The datasets of Quantitative Proteomic Analysis generated for this study were uploaded to MassIVE, and was assigned the dataset identifier MassIVE MSV000085952, which can be downloaded at ftp://massive.ucsd.edu/MSV000085952/.

## Author Contributions

JX and HL conceived and designed the experiments. HL, FK, C-YH, and XG performed the experiments. HL, FK, C-YH, PL, DS, and JX analyzed the data. HL and JX wrote the article with contributions of all the authors.

## Conflict of Interest

The authors declare that the research was conducted in the absence of any commercial or financial relationships that could be construed as a potential conflict of interest.

## Footnotes

^1^http://www.webgestalt.org/option.php

^2^http://kobas.cbi.pku.edu.cn/kobas3

^3^https://string-db.org/

^4^https://biit.cs.ut.ee/clustvis
